# Is there a negative impact of winter on mental distress and sleeping problems in the subarctic: The Tromsø Study

**DOI:** 10.1186/1471-244X-12-225

**Published:** 2012-12-12

**Authors:** May Trude Johnsen, Rolf Wynn, Trond Bratlid

**Affiliations:** 1 Department of Clinical Medicine, Faculty of Health Sciences, University of Tromsø, Tromsø, Norway; 2 University Hospital of North Norway, Tromsø, Norway

**Keywords:** Sleep, Polar day, Polar night, Seasonality, Mental distress, Insomnia, Sub-arctic

## Abstract

**Background:**

Prior studies have suggested that the darkness of winter impacts the level of mental distress and sleeping problems. Our study investigated whether people living in the sub-arctic had more sleeping problems or mental distress during winter.

**Methods:**

The cross sectional population Tromsø Study was conducted in Tromsø, North Norway, at 69.4 degrees North and above the Arctic Circle. The study included entire birth cohorts and random samples of the population aged 30 to 87 years. Data was collected continuously from 1 October 2007 to the end of December 2008 except July. 8951 persons completed questionnaires including the HSCL-10 and the MCTQ.

**Results:**

There were no significant differences in the reporting of current mental distress depending on season. Significantly more reported current sleeping problems in winter than in the other seasons, and less sleeping problems was found in spring.

**Conclusions:**

In this sub-arctic population, insomnia was most prevalent in winter, but there were no significant seasonal differences in mental distress. Although some people in the sub-arctic clearly are mentally negatively affected by the darkness of winter, the negative impact of winter on mental distress for the adult population is not conclusive.

## Background

Many studies have focused on seasonality of mental distress and psychiatric symptoms, and in 1984, Rosenthal et al. [1] first described the syndrome Seasonal Affective Disorder (SAD). The condition is known as ‘winter depression’ due to a predictable onset in the autumn/winter months and a spontaneous remission in the spring/summer period.

Chronobiological mechanisms related to circadian rhythms, melatonin, serotonin, and retinal photosensitivity have been claimed to play a significant role in many cases of seasonal mood variations because of the described linkage between the syndrome and the lack of daylight in winter [2-5].

Seasonal differences in daylight also influence sleep in many ways. The term ’insomnia’ is often defined as an individual’s report of difficulty with sleep or used to describe the presence of polysomnographic evidence of disturbed sleep. Insomnia symptoms are of the most frequent complaints in the general population with prevalences from 10–50% depending in part on definitions and data-collection methodologies [6,7].

The clinical symptoms of Midwinter Insomnia (MI) in Northern Norway, as described by Lingjaerde et al. [2] start at the beginning of the Polar night period (ultimo November) and last until the sun returns over the horizon (ultimo January). Cloudy and stormy weather may also play a part [8]. MI severity varies from moderate difficulties in falling asleep to almost total inability to sleep during the whole night. These symptoms stand in contrast to the hypersomnia associated with SAD, which often continues into the day, leading to excessive daytime somnolence [9].

Northern Norway is ideal for studying the seasonal influence of natural light on sleep and mental distress because of the seasonal differences in light exposure [10,11]. The first regular study of sleeping problems during the darkness season in northern Norway was performed in 1957 [12] among school pupils aged 15–22, showing that 38.7% complained of sleep difficulties from September to January, compared to 5.8% for a group living in South Norway. Later, several studies have reported various and partly conflicting results regarding the levels of insomnia or mental distress in the dark period. Husby and Lingjaerde found that midwinter insomnia was more common than insomnia in other seasons [13], and Haggag et al. found major seasonal mood variations [14]. Hansen et al. found no significant relationship between participation month and neither depression nor insomnia [15], and Partonen et al. suggested that a high latitude was not responsible for major depression with a seasonal pattern [16].

The objectives of this study were to investigate whether people living in the sub-arctic reported more mental distress or had more sleeping problems during winter than in other seasons.

## Methods

### Study area and study population

The modern university city of Tromsø is situated at 69.4 degrees North and above the Arctic Circle, and has about 70 000 inhabitants. In autumn there is a rapid change in daylight towards the end of November when the sun moves below the horizon line. After a two month period with very little daylight (the Polar night), the sun is back above the horizon in the second half of January. A rapid increase in daylight culminates in the second half of May in a two month long period when the sun is above the horizon 24 hours a day (the Polar day). This midnight sun period lasts until the end of July when the sun again begins to dip under the horizon for a longer period each night, and the cycle starts over again.

The 6th part of the cross sectional population ‘Tromsø Study’ included entire birth cohorts and random samples of the population of Tromsø aged 30 years or more [17]. The invited population consisted of four groups: Subjects who took part in the special study in the Tromsø IV survey, a 10% random sample of subjects aged 30–39, all individuals aged 40–42 or 60–87 and a 40% random sample of subjects aged 43–59 years. Data was collected continuously from 1 October 2007 to 19 December 2008 except during the summer holiday in July, and 12984 men and women participated. The total response rate was 65.7%, but lower in age group 30–34 years (44%) and in age group 75–87 years (51%). 87% of the study population defined themselves as Norwegian, 1.6% as Sami, 1.3% as Kven/Finnish and the rest as other/not answered. The study was approved by the Regional Medical Ethics Committee.

A questionnaire was mailed together with the invitation, including questions about socioeconomic situation, general health status, diseases, physical discomfort and mental distress, use of the public health services and medication, smoking and alcohol habits, and physical activity. Those who participated in the study received a second questionnaire, including questions related to symptoms of anxiety and depression (completed by 11 118 or 85% of the participants), and to sleep habits and sleep-wake rhythms (completed by 8 978 or 76.1% of the participants). We excluded data for participants where we had information about their sleep only during work days (n=27). This article describes analyses done for the 8 951 persons aged 30 to 87 years who had answered the questions relating to mental distress and to sleep habits.

### Variables

#### Sleep questionnaire

To examine sleeping habits, a Norwegian translation of The Munich ChronoType Questionnaire (MCTQ) was used [18]. The questionnaire included 15 items, with questions about the number of work days and free time during the week, shift work, and bedtime and rise time including sleep latency (in minutes) during work days and days off work. Sleep duration on work days and free days was calculated as wake up time minus (‘the time I get ready to fall asleep’ plus ‘sleep latency’). To quantify mean sleep duration, we calculated sleep duration on work days x number of work days plus sleep duration on free days x number of free days/7. In addition to the MCTQ, questions regarding sleeplessness during the last 12 months (never or just a few times a year, 1–3 times a month, approximately once a week, or more than once a week) and difficulties sleeping during the last two weeks (not at all, no more than usual, rather more than usual or much more than usual) were answered. If they suffered from sleeplessness monthly or more often, they were asked to state which time of year (winter, summer, spring, autumn, or no particular season).

#### HSCL-10

The Hopkins Symptoms Checklist is a screening instrument for detecting mental distress, commonly used in population studies [19]. The 90 items HSCL-90 has been reduced into various versions, and the validated 10-item version (HSCL-10) is almost as good as the longest version in detecting mental distress in the general population [20]. In an earlier Norwegian population study [21], the established cut-off for HSCL-10 yielded a prevalence rate of 11.4% and a mean score of 1.85 or higher has been found to indicate high mental distress.

Symptoms of mental distress during the last week before admission were measured using the HSCL-10; Have you during the last week: Experienced sudden fear without apparent reason, Felt afraid or worried, Experienced faintness or dizziness, Been tense or upset, Easily blamed yourself, Experienced sleeplessness, Felt depressed or sad, Felt that your life is a struggle, Felt hopelessness with regard to the future? The answers were given in 4 categories (No, A little, Pretty much, A lot), and the mean HSCL-10 score was calculated by dividing the total score with ten (number of items). Individuals with missing values have been excluded in the mean item score calculation.

#### Season

The participants attended the study during all months except July. Because of the Polar night from November to January and the Polar day from May to July, we chose to divide the year into 4 seasons: winter (November, December, January), spring (February, March, April), summer (May, June, July), and autumn (August, September, October).

#### Analyses

The analyses were performed with STATA version 11.1 (Stata/SE 12.0 for Windows, StataCorp LP, College Station, TX, USA). Age was grouped in four intervals: 35–44, 45–54, 55–64 and 65–87 years.

Univariate analyses were conducted by one-way ANOVA followed by the Scheffe post hoc test or Kruskal-Wallis test. First we did the univariate analyses between season of participation and: 1) sleep duration, 2) different sleep variables, 3) the use of sleeping pills, and 4) HSCL-10 score. Subsequently, stepwise backwards multiple logistic regression were performed, with ‘more sleeping problems than usual during the last couple of weeks’ and HSCL-10 score as outcome variables, and with participation season, socioeconomic variables (age, sex, interaction variable “age*sex”, educational level, living with spouse), lifestyle variables (current smoking, alcohol habits, physical activity) and health variables (coping, self –rated physical health, use of painkillers, and HSCL-10 score in the calculation of sleeping problems) as independent variables. Because we collected data from each participant only once, the seasonal variations were based on participation date, and not individual variations. The statistical significance level was set at 0.05.

## Results

### Sample characteristics

Mean population age was 54.8 years (range 30–87 years, SD 11.8), and 4 610 (51.5%) of the participants were women. 33.6% of the attendees participated in the study in winter, 20.4% in spring, 15.1% in summer, and 30.9% in autumn.

### Sleep

Mean sleep duration was 7 hours (h) 2 minutes (m) (SD=0.96) for men and 7 h 15 min (SD=1.01) for women. 5828 persons (66.0%) reported that they had not suffered from sleeplessness during the last 12 months. 8061 persons (92.1%) had not used sleeping pills during the last four weeks before attending the study, and 400 persons (4.6%) reported having used sleeping pills every week/daily during the last four weeks.

1557 persons (17.4%) reported suffering from sleeplessness monthly or more often related to a specific season. 1088 of these (69.9%) reported Polar night (midwinter) sleeplessness, 245 (15.7%) reported sleeplessness in the midnight sun period, and 224 (14.4%) reported the same in spring or autumn. Stratifying the population by season of participation (Table 1) showed that among those attending the study in winter, 14% reported Polar night sleeplessness. Among those attending the study in summer, about 10% reported Polar night sleeplessness (Kruskal-Wallis test: df=3, chi-squared=11.78, p=0.008).

**Table 1:** Seasonal differences in sleep

	**Season for attending the study**				
	**Polar night**	**Spring**	**Polar Day**	**Autumn**	**p**^**3**^
**Mean sleep duration**	7.12 (0.96)^1^	7.17 (1.00)^1^	7.15 (1.01)^1^	7.14 (1.01)^1^	0.20
**Usually suffering from sleeplessness during Polar night**	420 (14.0)^2^	261 (14.3)^2^	136 (10.1)^2^	271 (9.8) ^2^	0.008
**Current sleeping problems**	278 (9.4)^2^	79 (4.4)^2^	74 (5.6)^2^	134 (4.9)^2^	<0.001
**-rather more than usual**	223 (7.5)	69 (3.8)	62 (4.7)	116 (4.3)	<0.001
**-much more than usual**	55 (1.9)	10 (0.6)	12 (0.9)	18 (0.7)	<0.001

^1^Mean sleep duration in hours (SD).

^2^Number (%).

^3^Kruskal-Wallis test

By using a backwards stepwise logistic regression, we found that the odds of reporting sleeplessness during Polar night were over 50% higher among winter attendees compared to summer attendees (Logistic regression: OR=1.56, z=3.51, p<0.001, 95% CI=1.22-2.01).

We found seasonal differences in current sleeping problems (‘more sleeping problems than usual during the last couple of weeks’) (Kruskal-Wallis test, df=3, chi-squared=67.16, p<0.001). The highest rate of current sleeping problems was reported among winter attendees, who also more often reported “much more sleeping problems than usual” than did attendees in other seasons. The odds of sleeping problems for winter attendees was about 80% higher than for summer attendees (Logistic regression: OR=1.86, p<0.001, 95% CI=1.52-2.29). The results of the logistic regression model are shown in Table 2.

**Table 2:** Factors related to current sleeping problems and high mental distress

	**Current sleeping problems**				**High mental distress**			
	**OR**^**1**^	**SE**	**z**	**p**	**OR**^**1**^	**SE**	**z**	**p**
**Male**^**2**^				Ns^3^	0.66	0.07	−3.84	<0.001
**Age**^**4**^				Ns^3^	0.98	0.005	−4.72	<0.001
Age*Sex^**5**^	0.99	0.002	−5.36	<0.001				Ns^3^
**Education**^**6**^	1.72	0.19	4.99	<0.001				Ns^3^
**Living with spouse**^**7**^				Ns^3^	0.47	0.05	−6.77	<0.001
**Smoker**^**8**^				Ns^3^				Ns^3^
**Alcohol frequency**^**9**^				Ns^3^				Ns^3^
**Alcohol units**^**10**^				Ns^3^				Ns^3^
**Exercise**^**11**^				Ns^3^				Ns^3^
**Season**^**12**^	1.86	0.20	5.91	<0.001				Ns^3^
**Painkillers**^**13**^				Ns^3^	1.94	0.23	5.57	<0.001
**Health**^**14**^	0.70	0.08	−3.01	0.003	0.18	0.02	−15.57	<0.001
**Coping**^**15**^				Ns^3^				Ns^3^
**High mental distress**^**16**^	4.95	0.68	11.67	<0.001	-	-	-	-
**Sleeping problems**	-	-	-	-	4.62	0.64	11.12	<0.001
**Constant**	0.05	0.01	−22.46	<0.001	0.94	0.27	−0.21	0.835

The table shows the results of stepwise logistic regression with “more sleeping problems than usual during the last couple of weeks” and HSCL-10≥1.85 as dependent variables.

^1^Odds ratio.

^2^Males compared to females.

^3^Not significant.

^4^Change per year of age.

^5^Interaction variable “Age*Sex”.

^6^Educational level: Primary school vs. education beyond primary school.

^7^Living with spouse compared to living alone.

^8^Current smoking compared to not smoking.

^9^Alcohol frequency: 2–3 times per month or more compared to monthly or less.

^10^1-4 units of alcohol per drinking episode compared to 5 units or more.

^11^Exercise 2 hours per week or more, compared to once per week or less.

^12^Change between seasons: Winter compared to summer.

^13^Use of any kind of painkillers weekly or more in the last four weeks.

^14^General considering of own health (excellent, good, neither good nor bad, bad, very bad).

^15^Felt unable to cope with difficulties during the past couple of weeks (not at all, no more than usual, rather more than usual, much more than usual).

^16^HSCL10 score≥1.85.

The use of sleeping pills was highest in summer (6.0%) and lowest in spring (4.2%), but the difference was not significant.

### Mental distress

8010 persons (89.5% of our study population) had complete data for the HSCL-10 questionnaire. Mean score for the population was 1.27 (SD 0.36). High mental distress (HSCL-10≥1.85) was found for 590 persons (7.4%). As shown in Table 3, summer and autumn attendees had the highest HSCL-10 scores, but the seasonal differences were not significant when comparing attendees in all four seasons. We also found that the proportion of mental distress above the cut off level (HSCL-10≥1.85) was lowest among spring attendees and highest among summer attendees. These seasonal differences were not significant.

**Table 3:** Seasonal differences in mental distress

	**Season for attending the study**					
	**Polar night**	**Spring**	**Polar day**	**Autumn**	**χ2**^**1**^	**p**^**1**^
**HSCL-10 score**	1.26 (0.36)^2^	1.25 (0.35)^2^	1.27 (0.37)^2^	1.27 (0.37)^2^	5.514	0.3189
**High mental distress**^**3**^	206 (7.7)^4^	107 (6.6)^4^	97 (8.0)^4^	180 (7.3)^4^	2.767	0.420

^1^Kruskal-Wallis test.

^2^Mean (SD).

^3^HSCL10 score≥1.85.

^4^Number (%).

Table 2 summarizes the logistic regression model with HSCL-10≥1.85 as the dependent variable.

## Discussion

Our findings support the idea that the negative impact of winter on mental distress is –for the majority of the adult population- more a myth than a fact, or at least difficult to substantiate. 7.4% of the study population had a high level of mental distress. This is lower than the average for the general population in Norway (11.4%) [21]. There were no significant differences in the reporting of current mental distress depending on season. Asking for current sleeping problems and current symptoms of mental distress lowers the risk of memory distortion and recall bias, which is one of the problems with retrospective studies [15,22]. In our study, mental distress appeared to be a stable phenomenon independent of season and the extreme variations in light and darkness found in the sub-arctic. One could expect that season should be of importance to the level of mental distress, i.e. as in SAD. Moreover, one would expect a higher prevalence of sufferers of SAD at higher latitudes [14,23,24]. There seems to be less correlation between prevalence rates of SAD and latitude than expected [25,26], and other factors like climate, genetic vulnerability and socio–cultural context may play a more important role [27]. Saarijarvi et al. [28] found that the prevalence of overall and winter SAD did not differ between Finns living in northern and south-western Finland and that winter SAD was less frequent among the Sami. They suggest a genetic selection effect which could lead to better tolerance of the arctic environment.

The polar night in Tromsø is not completely dark, and the outdoor daylight intensity (usually increased by reflecting snow) on bright days at the beginning and the end of the dark period may be strong enough to minimize the seasonal effects on mood. However, we do not know whether our studied subgroup may be extra resistant to seasonal variations in light exposure.

The winter attendees in our study reported significantly more current sleeping problems than the attendees in other seasons. This corresponds to the finding of a different recent study involving people in the sub-arctic [29]. It is possible that latitude is of importance to the prevalence of sleeping problems, as the same study did not find such problems in a sample of people living at 5 degrees North. Moreover, in a Norwegian study south of the Polar circle (at 63°-65° N), no evidence of a seasonal variation on reports of insomnia symptoms or time in bed was found [30].

Drawing on these findings, it is possible to hypothesise that seasonal variations in light-darkness are of greater importance to the prevalence of sleeping problems than to the prevalence of mental distress.

There are some important limitations in this study that should be mentioned. First, our study is limited to the adult population. We know from other studies that the level of mental distress is higher among adolescents, young adults and the elderly [21]. Our study did not include persons below 30, and the quite low response rate for the youngest and oldest age groups could lead to an underestimation of the level of mental distress. However, we have no reason to believe that the age span in our study would mask any seasonal differences in mental distress.

Second, there may be a selection bias in that non-attendees may actually suffer from more mental distress and sleeping problems than those who attended the study. Hansen et al. found that non-attendees to a general health survey had more than twice the risk of attendees of having a psychiatric disorder [31]. Knudsen et al. found that the risk of being a recipient of disability pension for mental disorders was three times higher among non-attendees [32]. Sivertsen et al. found that insomnia was an independent risk factor for long term sick leave [33]. If we transfer these findings to our study, there is a possibility that some people with high mental distress or severe sleeping problems during winter were too affected to participate in the study. However, none of these studies showed seasonal differences in participation rates related to mental distress or sleeping problems. Unfortunately, we had no data for attendance rates by month or season in our study.

A third limitation is that our data were collected at one single occasion, so the seasonal variations were based on the recordings at the different attending dates.

In a population study like the present, there is a possibility of reporting bias. A fourth limitation is that the attendees were not clearly blinded to the research questionnaire, as the statement of which time of year they suffered from sleeplessness could lead to a willingness to confirm the general myth. Although a higher proportion of individuals reported insomnia when queried in the winter, the differences did not appear to be as large as could be expected. This possible reporting bias could be a significant factor that might account for the different findings of the Nord-Trøndelag survey [30] and the Tromsø study.

One strength of the present study is the relatively high number of attendees. A high n makes it easier to detect any patterns in mental distress and sleeping problems. The overall response rate was 65.7%, which is relatively high for this type of study and which also ensures that the findings were representative of the population. However, the response rates for the young and the elderly were somewhat below the average, which could be a source of bias for these sub-groups.

Another strength of the Tromsø study in general is that it covered a variety of topics, and questions about mood and sleep were only a minor part of the large questionnaire. There was no focus on mental distress or sleep when information about the study was mentioned in the local newspapers. This could minimize the possibility of selection bias.

## Conclusions

The present study showed no significant seasonal differences in the prevalence of current mental distress. Sleeping problems were most prevalent in winter and less in spring. Although some people in the sub-arctic clearly are mentally negatively affected by the darkness of winter, this was not confirmed by the participants in the Polar night period. Further longitudinal studies on larger subgroups of the population should be done to investigate the impact of winter on mental distress and sleeping problems.

## Competing interests

This was not an industry supported study. The authors have indicated no conflicts of interest.

## Authors’ contributions

TB contributed in designing the study, wrote the protocol and contributed to the literature searches. MTJ managed the literature searches and analyses, undertook the statistical analysis and wrote the first draft of the manuscript. RW contributed to the statistical analysis and the English language translation. All authors revised the manuscript and approved the final manuscript.

## Pre-publication history

The pre-publication history for this paper can be accessed here:

http://www.biomedcentral.com/1471-244X/12/225/prepub
